# Supplementary material for: Local guidelines for admission to UK midwifery units compared with national guidance: A national survey using the UK Midwifery Study System (UKMidSS)
Source: PLoS One. 2020 Oct 20;15(10):e0239311. doi: 10.1371/journal.pone.0239311 (PMC7575094; doi:10.1371/journal.pone.0239311)
Supplement: S1 File — (DOCX) [file pone.0239311.s001.docx]

**Criteria to be assessed for women planning place of birth**

Tables 1-4, below reproduce Tables 6-9 from national guidance, NICE CG190: Intrapartum care for healthy women and babies (pp 11-15), indicating specific criteria to be assessed for women planning place of birth.

**Table 1. Medical conditions indicating increased risk suggesting planned birth in an obstetric unit (Table 6 in NICE CG190)**

| **Disease area** | **Medical condition** |
| --- | --- |
| Cardiovascular | Confirmed cardiac disease  Hypertensive disorders |
| Respiratory | Asthma requiring an increase in treatment or hospital treatment  Cystic fibrosis |
| Haematological | Haemoglobinopathies – sickle‑cell disease, beta‑thalassaemia major  History of thromboembolic disorders  Immune thrombocytopenia purpura or other platelet disorder or platelet count below 100×10^9^/litre  Von Willebrand's disease  Bleeding disorder in the woman or unborn baby  Atypical antibodies which carry a risk of haemolytic disease of the newborn |
| Endocrine | Hyperthyroidism  Diabetes |
| Infective | Risk factors associated with group B streptococcus whereby antibiotics in labour would be recommended |
| Immune | Systemic lupus erythematosus  Scleroderma |
| Renal | Abnormal renal function  Renal disease requiring supervision by a renal specialist |
| Neurological | Epilepsy  Myasthenia gravis  Previous cerebrovascular accident |
| Gastrointestinal | Liver disease associated with current abnormal liver function tests |
| Psychiatric | Psychiatric disorder requiring current inpatient care |

Source: National Institute for Health and Care Excellence. Intrapartum care for healthy women and babies: Clinical Guideline CG190. London: National Institute for Health and Care Excellence; 2014.

**Table 2. Other factors indicating increased risk suggesting planned birth in an obstetric unit (Table 7 in NICE CG190)**

| **Factor** | **Additional information** |
| --- | --- |
| Previous complications | Unexplained stillbirth/neonatal death or previous death related to intrapartum difficulty  Previous baby with neonatal encephalopathy  Pre‑eclampsia requiring preterm birth  Placental abruption with adverse outcome  Eclampsia  Uterine rupture  Primary postpartum haemorrhage requiring additional treatment or blood transfusion  Retained placenta requiring manual removal in theatre  Caesarean section  Shoulder dystocia |
| Current pregnancy | Multiple birth  Placenta praevia  Pre‑eclampsia or pregnancy‑induced hypertension  Preterm labour or preterm prelabour rupture of membranes  Placental abruption  Anaemia – haemoglobin less than 85 g/litre at onset of labour  Confirmed intrauterine death  Induction of labour  Substance misuse  Alcohol dependency requiring assessment or treatment  Onset of gestational diabetes  Malpresentation – breech or transverse lie  BMI at booking of greater than 35 kg/m^2^  Recurrent antepartum haemorrhage  Small for gestational age in this pregnancy (less than fifth centile or reduced growth velocity on ultrasound)  Abnormal fetal heart rate/doppler studies  Ultrasound diagnosis of oligo‑/polyhydramnios |
| Previous gynaecological history | Myomectomy  Hysterotomy |

Source: National Institute for Health and Care Excellence. Intrapartum care for healthy women and babies: Clinical Guideline CG190. London: National Institute for Health and Care Excellence; 2014.

**Table 3. Medical conditions indicating individual assessment when planning place of birth (Table 8 in NICE CG190)**

| **Disease area** | **Medical condition** |
| --- | --- |
| Cardiovascular | Cardiac disease without intrapartum implications |
| Respiratory | Asthma requiring an increase in treatment or hospital treatment  Cystic fibrosis |
| Haematological | Atypical antibodies not putting the baby at risk of haemolytic disease  Sickle cell trait  Thalassaemia trait  Anaemia – haemoglobin 85–105 g/litre at onset of labour |
| Endocrine | Unstable hypothyroidism such that a change in treatment is required |
| Infective | Hepatitis B/C with normal liver function tests |
| Immune | Non specific connective tissue disorders |
| Skeletal/Neurological | Spinal abnormalities  Previous fractured pelvis  Neurological deficits |
| Gastrointestinal | Liver disease without current abnormal liver function  Crohn's disease  Ulcerative colitis |

Source: National Institute for Health and Care Excellence. Intrapartum care for healthy women and babies: Clinical Guideline CG190. London: National Institute for Health and Care Excellence; 2014.

**Table 4. Other factors indicating individual assessment when planning place of birth (Table 9 in NICE CG190)**

| **Factor** | **Additional information** |
| --- | --- |
| Previous complications | Stillbirth/neonatal death with a known non recurrent cause  Pre eclampsia developing at term  Placental abruption with good outcome  History of previous baby more than 4.5 kg  Extensive vaginal, cervical, or third or fourth degree perineal trauma  Previous term baby with jaundice requiring exchange transfusion |
| Current pregnancy | Antepartum bleeding of unknown origin (single episode after 24 weeks of gestation)  BMI at booking of 30–35 kg/m2  Blood pressure of 140 mmHg systolic or 90 mmHg diastolic or more on 2 occasions  Clinical or ultrasound suspicion of macrosomia  Para 4 or more  Recreational drug use  Under current outpatient psychiatric care  Age over 35 at booking |
| Fetal indications | Fetal abnormality |
| Previous gynaecological history | Major gynaecological surgery  Cone biopsy or large loop excision of the transformation zone  Fibroids |

Source: National Institute for Health and Care Excellence. Intrapartum care for healthy women and babies: Clinical Guideline CG190. London: National Institute for Health and Care Excellence; 2014.
