# Supplementary material for: Local guidelines for admission to UK midwifery units compared with national guidance: A national survey using the UK Midwifery Study System (UKMidSS)
Source: PLoS One. 2020 Oct 20;15(10):e0239311. doi: 10.1371/journal.pone.0239311 (PMC7575094; doi:10.1371/journal.pone.0239311)
Supplement: S2 File — (DOCX) [file pone.0239311.s002.docx]

**Characteristics of responding and non-responding maternity services**

**Table 1. Characteristics of responding and non-responding organisations (sent guideline)**

|  | | **Sent guideline** | | | |  |
| --- | --- | --- | --- | --- | --- | --- |
|  | | **Yes** | **%** | **No** | **%** | ***p*-value** |
| **Configuration of care** | |  |  |  |  |  |
| AMU |  | 52 | 72.2 | 20 | 27.8 |  |
| FMU |  | 9 | 56.3 | 7 | 43.8 |  |
| AMU & FMU |  | 26 | 76.5 | 8 | 23.5 |  |
| All |  | 87 | 71.3 | 35 | 28.7 | 0.325 |
| **Number of births per year^a^** | |  |  |  |  |  |
| <3,500 |  | 20 | 60.6 | 13 | 39.4 |  |
| 3,500-4,999 |  | 20 | 74.1 | 7 | 25.9 |  |
| 5,000-5,999 |  | 22 | 75.9 | 7 | 24.1 |  |
| 6,000-17,000 |  | 25 | 75.8 | 8 | 24.2 | 0.464 |
| **% of AMU births per year^b^** | |  |  |  |  |  |
| 0-10 |  | 14 | 53.9 | 12 | 46.2 |  |
| 10.1-15 |  | 20 | 76.9 | 6 | 23.1 |  |
| 15.1-20 |  | 30 | 79.0 | 8 | 21.0 |  |
| 20.1-39 |  | 17 | 73.9 | 6 | 26.1 | 0.237 |

^a^ Overall annual number of births in the maternity service (NHS Trust or Health Board)

^b^ Data missing for nine maternity services

**Table 2. Characteristics of responding and non-responding maternity services (completed survey).**

|  | | **Completed Survey** | | | |  |
| --- | --- | --- | --- | --- | --- | --- |
|  |  | **Yes** | **%** | **No** | **%** | ***p*-value** |
| **Configuration of care** | |  |  |  |  |  |
| AMU |  | 65 | 90.3 | 7 | 9.7 |  |
| FMU |  | 9 | 56.3 | 7 | 43.7 |  |
| AMU & FMU |  | 28 | 82.4 | 6 | 17.7 | 0.004 |
| **Number of births per year^a^** | |  |  |  |  |  |
| <3,500 |  | 26 | 78.8 | 7 | 21.2 |  |
| 3,500-4,999 |  | 22 | 81.5 | 5 | 18.5 |  |
| 5,000-5,999 |  | 27 | 93.1 | 2 | 6.9 |  |
| 6,000-17,000 |  | 27 | 81.8 | 6 | 18.2 | 0.452 |
| **% of AMU births per year^b^** | |  |  |  |  |  |
| 0-10 |  | 18 | 69.2 | 8 | 30.8 |  |
| 10.1-15 |  | 23 | 88.5 | 3 | 11.5 |  |
| 15.1-20 |  | 36 | 94.7 | 2 | 5.3 |  |
| 20.1-39 |  | 20 | 87.0 | 3 | 13.0 | 0.011 |

^a^ Overall annual number of births in the maternity service (NHS Trust or Health Board)

^b^ Data missing for nine maternity services
