# Supplementary material for: Local guidelines for admission to UK midwifery units compared with national guidance: A national survey using the UK Midwifery Study System (UKMidSS)
Source: PLoS One. 2020 Oct 20;15(10):e0239311. doi: 10.1371/journal.pone.0239311 (PMC7575094; doi:10.1371/journal.pone.0239311)
Supplement: S3 File — (DOCX) [file pone.0239311.s003.docx]

Individual admission criteria that were ‘more inclusive’ than NICE CG190 are listed in Tables 1-3

**Table 1. ‘More inclusive’ criteria - NICE CG190 recommends individual assessment before admission, but midwifery units will explicitly admit women with these conditions/risk factors**

| **Criteria** | **Guidelines in which criteria were listed** | | | |
| --- | --- | --- | --- | --- |
|  | **n** | | **%^a^** | |
| **Medical conditions** |  | |  | |
| Outpatient psychiatric care | 5 |  | 6.8 |  |
| Atypical antibodies | 4 |  | 5.5 |  |
| Sickle cell/Thalassaemia trait | 4 |  | 5.5 |  |
| Medical condition not impacting on pregnancy | 4 |  | 5.5 |  |
| Hepatitis B/C, normal LFTs | 2 |  | 2.7 |  |
| **Obstetric history** |  |  |  |  |
| Previous 3^rd^ degree tear | 10 |  | 13.7 |  |
| Previous 4^th^ degree tear | 4 |  | 5.5 |  |
| Previous stillbirth, non-recurrent cause | 2 |  | 2.7 |  |
| Previous baby >4.5kg | 1 |  | 1.4 |  |
| Previous pre-eclampsia | 1 |  | 1.4 |  |
| **Current pregnancy** |  |  |  |  |
| Parity ≥4 | 68 |  | 93.2 |  |
| Maternal age 35-40yrs | 54 |  | 74.0 |  |
| BMI 30-35kg/m^2^ | 47 |  | 64.4 |  |
| Fetal abnormality | 1 |  | 1.4 |  |
| Recreational drug use | 1 |  | 1.4 |  |

^a^ Percentage of the 73 guidelines that contained at least one criterion that was ‘more inclusive’ than NICE CG190

**Table 2. ‘More inclusive’ criteria - NICE CG190 recommends obstetric unit but midwifery unit will individually assess women with these conditions/risk factors**

| **Criteria** | **Guidelines in which criteria were listed** | | | |
| --- | --- | --- | --- | --- |
|  | **n** | | **%^a^** | |
| **Medical conditions** |  | |  | |
| Group B Streptococcus colonisation | 16 |  | 21.9 |  |
| Gestational diabetes | 15 |  | 20.5 |  |
| Induction of labour, one intervention | 13 |  | 17.8 |  |
| HIV positive, undetectable viral load | 11 |  | 15.1 |  |
| Infection (chicken pox) | 1 |  | 1.4 |  |
| **Obstetric history** |  |  |  |  |
| Previous manual removal of placenta | 13 |  | 17.8 |  |
| Previous shoulder dystocia | 10 |  | 13.7 |  |
| Previous caesarean section | 3 |  | 4.1 |  |
| **Current pregnancy** |  |  |  |  |
| BMI 35-40kg/m^2^ | 6 |  | 8.2 |  |
| Multiparous, BMI 35-40kg/m^2^ | 5 |  | 6.8 |  |
| Previous stillbirth or neonatal death | 1 |  | 1.4 |  |
| Substance misuse | 1 |  | 1.4 |  |

^a^ Percentage of the 73 guidelines that contained at least one criterion that was more inclusive than NICE CG190

**Table 3. ‘More inclusive’ criteria - NICE CG190 recommends obstetric unit but midwifery unit will explicitly accept women with these conditions/risk factors**

| **Criteria** | **Guidelines in which criteria were listed** | | | |
| --- | --- | --- | --- | --- |
|  | **n** | | **%^a^** | |
| **Medical conditions** |  | |  | |
| Group B Streptococcus colonisation | 21 |  | 28.8 |  |
| Induction of labour, one intervention | 21 |  | 28.8 |  |
| HIV positive, undetectable viral load | 4 |  | 5.5 |  |
| Gestational diabetes | 2 |  | 2.7 |  |
| **Obstetric history** |  |  |  |  |
| Previous manual removal of placenta | 2 |  | 2.7 |  |
| Previous shoulder dystocia | 1 |  | 1.4 |  |
| **Current pregnancy** |  |  |  |  |
| BMI 35-40kg/m^2^ | 10 |  | 13.7 |  |
| Multiparous, BMI 35-40kg/m^2^ | 16 |  | 13.7 |  |

^a^ Percentage of the 73 guidelines that contained at least one criterion that was more inclusive than NICE CG190
