# Supplementary material for: Local guidelines for admission to UK midwifery units compared with national guidance: A national survey using the UK Midwifery Study System (UKMidSS)
Source: PLoS One. 2020 Oct 20;15(10):e0239311. doi: 10.1371/journal.pone.0239311 (PMC7575094; doi:10.1371/journal.pone.0239311)
Supplement: S4 File — (DOCX) [file pone.0239311.s004.docx]

Individual admission criteria that were ‘more restrictive’ than NICE CG190 are listed in Tables 1-3

**Table 1. ‘More restrictive’ criteria – NICE CG190 recommends individual assessment, but midwifery unit will explicitly not admit women with these conditions/risk factors**

| **Criteria** | **Guidelines in which criteria were listed** | | | |
| --- | --- | --- | --- | --- |
|  | **n** | | **%^a^** | |
| **Medical conditions** |  | |  | |
| Inflammatory bowel disease | 4 |  | 5.6 |  |
| Blood pressure >140/90, on two occasions | 2 |  | 2.8 |  |
| **Obstetric history** |  |  |  |  |
| Previous baby >4.5kg | 16 |  | 22.5 |  |
| Previous 4^th^ degree tear | 3 |  | 4.2 |  |
| Previous 3^rd^ degree tear | 1 |  | 1.4 |  |
| **Current pregnancy** |  |  |  |  |
| Fetal abnormality | 4 |  | 5.6 |  |
| Other social factors | 2 |  | 2.8 |  |

^a^ Percentage of the 71 guidelines that contained at least one criterion that was more restrictive than NICE CG190

**Table 2. ‘More restrictive’ criteria – conditions/risk factors not mentioned by NICE CG190, but for which midwifery unit explicitly recommends individual assessment before considering admission**

| **Criteria** | **Guidelines in which criteria were listed** | | | |
| --- | --- | --- | --- | --- |
|  | **n** | | **%^a^** | |
| **Medical conditions** |  | |  | |
| Declining blood products | 15 |  | 21.1 |  |
| Anaesthetic problems | 5 |  | 7.0 |  |
| Malignant disease | 2 |  | 2.8 |  |
| Blood pressure >140/90, on one occasion | 1 |  | 1.4 |  |
| **Obstetric history** |  |  |  |  |
| Assisted conception | 6 |  | 8.5 |  |
| Recurrent miscarriage (x3) | 5 |  | 7.0 |  |
| IVF, donor gametes | 4 |  | 5.6 |  |
| Previous difficult instrumental birth | 2 |  | 2.8 |  |
| **Current pregnancy** |  |  |  |  |
| Female genital cutting | 18 |  | 25.4 |  |
| Late booking/no antenatal care | 16 |  | 22.5 |  |
| Other social factors | 14 |  | 19.7 |  |
| BMI <18kg/m^2^ | 12 |  | 16.9 |  |
| Maternal age <16yrs | 11 |  | 15.5 |  |
| Reduced fetal movements | 10 |  | 14.1 |  |
| Maternal age <18yrs | 8 |  | 11.3 |  |
| Child protection plan | 7 |  | 9.9 |  |
| Small for gestational age, <10^th^ centile | 4 |  | 5.6 |  |
| Obstetric cholestasis | 4 |  | 5.6 |  |
| Insignificant meconium | 1 |  | 1.4 |  |
| Low PAPP-A | 1 |  | 1.4 |  |

^a^ Percentage of the 71 guidelines that contained at least one criterion that was more restrictive than NICE

**Table 3. ‘More restrictive’ criteria – not mentioned by NICE CG190, but midwifery unit will explicitly not admit women with these conditions/risk factors**

| **Criteria** | **Guidelines in which criteria were listed** | | | |
| --- | --- | --- | --- | --- |
|  | **n** | | **%^a^** | |
| **Medical conditions** |  | |  | |
| Declining blood products | 11 |  | 15.5 |  |
| Anaesthetic problems | 4 |  | 5.6 |  |
| Blood pressure >140/90, on one occasion | 2 |  | 2.8 |  |
| Malignant disease | 1 |  | 1.4 |  |
| **Obstetric history** |  |  |  |  |
| IVF, donor gametes | 7 |  | 9.9 |  |
| Recurrent miscarriage (x3) | 2 |  | 2.8 |  |
| **Current pregnancy** |  |  |  |  |
| Maternal age <16yrs | 12 |  | 16.9 |  |
| Small for gestational age, <10^th^ centile | 9 |  | 12.7 |  |
| BMI <18kg/m^2^ | 7 |  | 9.9 |  |
| Late booking/no antenatal care | 7 |  | 9.9 |  |
| Female genital cutting | 6 |  | 8.5 |  |
| Child protection plan | 4 |  | 5.6 |  |
| Reduced fetal movements | 4 |  | 5.6 |  |
| Other social factors | 2 |  | 2.8 |  |
| Insignificant meconium | 1 |  | 1.4 |  |
| Maternal age <18yrs | 1 |  | 1.4 |  |

^a^ Percentage of the 71 guidelines that contained at least one criterion that was more restrictive than NICE
