# Supplementary material for: Local guidelines for admission to UK midwifery units compared with national guidance: A national survey using the UK Midwifery Study System (UKMidSS)
Source: PLoS One. 2020 Oct 20;15(10):e0239311. doi: 10.1371/journal.pone.0239311 (PMC7575094; doi:10.1371/journal.pone.0239311)
Supplement: S1 Table — (DOCX) [file pone.0239311.s005.docx]

**S1 Table. Number of midwifery units of each type in each country of the UK at the time of the survey**

|  | **AMU** | | **FMU** | | **All** | |
| --- | --- | --- | --- | --- | --- | --- |
| **Country** |  |  |  |  |  |  |
| England | 103 |  | 58 |  | 161 |  |
| Wales | 11 |  | 14 |  | 25 |  |
| Scotland | 6 |  | 15 |  | 21 |  |
| Northern Ireland | 6 |  | 3 |  | 9 |  |
| **Total UK** | **126** |  | **90** |  | **216** |  |

AMU: Alongside midwifery unit

FMU: Freestanding midwifery unit
