# Supplementary material for: Local guidelines for admission to UK midwifery units compared with national guidance: A national survey using the UK Midwifery Study System (UKMidSS)
Source: PLoS One. 2020 Oct 20;15(10):e0239311. doi: 10.1371/journal.pone.0239311 (PMC7575094; doi:10.1371/journal.pone.0239311)
Supplement: S2 Table — (DOCX) [file pone.0239311.s006.docx]

**S2 Table. Maternity service alongside midwifery unit (AMU) admission policy (opt-in or opt-out) by characteristics of maternity service**

|  | | **AMU opt-in** | | **AMU opt-out** | |  |
| --- | --- | --- | --- | --- | --- | --- |
|  |  | **n** | **%** | **n** | **%** | **p-value** |
| **Configuration of care** | | |  |  |  |  |
| AMU only |  | 20 | 31.2 | 44 | 68.8 |  |
| AMU & FMU |  | 10 | 35.7 | 18 | 64.3 | 0.674 |
| **Longest length of time AMU open** | | | |  |  |  |
| <3yrs |  | 0 | 0.0 | 3 | 100 |  |
| 3-5yrs |  | 7 | 50.0 | 7 | 50.0 |  |
| 6-10yrs |  | 10 | 30.3 | 23 | 69.7 |  |
| >10yrs |  | 13 | 31.7 | 28 | 68.3 | 0.412 |
| **Number of births^a^** | |  |  |  |  |  |
| <3,500 |  | 7 | 36.8 | 12 | 63.2 |  |
| 3,500-4,999 |  | 6 | 28.6 | 15 | 71.4 |  |
| 5,000-5,999 |  | 11 | 42.3 | 15 | 57.7 |  |
| 6,000-17,000 |  | 6 | 23.1 | 20 | 76.9 | 0.476 |
| **% AMU births** | |  |  |  |  |  |
| <10% |  | 5 | 50.0 | 5 | 50.0 |  |
| 10.1-15% |  | 8 | 34.8 | 15 | 65.2 |  |
| 15.1-20% |  | 14 | 38.9 | 22 | 61.1 |  |
| 20.1-39% |  | 2 | 10.5 | 17 | 89.5 | 0.100 |

^a^ Overall annual number of births in the maternity service (NHS Trust or Health Board)
